# Network meta-analysis on the efficacy and safety of finerenone versus SGLT2 inhibitors on reducing new-onset of atrial fibrillation in patients with type 2 diabetes mellitus and chronic kidney disease

**DOI:** 10.1186/s13098-022-00929-3

**Published:** 2022-10-27

**Authors:** Yaofu Zhang, Junheng Wang, Li Jiang, Tongxin Wang, Zhuang Li, Xiaozhe Fu, Weijun Huang, Yonghua Xiao, Shidong Wang, Jinxi Zhao

**Affiliations:** 1 Dongzhimen Hospital, Beijing University of Chinese Medicine, Beijing, China; 2 National Clinical Research Center for Chinese Medicine Cardiology, Xiyuan Hospital, China Academy of Chinese Medical Sciences, Beijing, China; 3 Key Laboratory of Chinese Internal Medicine of Ministry of Education and Beijing, Dongzhimen Hospital affiliated to Beijing University of Chinese Medicine, Beijing, China

**Keywords:** Atrial fibrillation, SGLT2 inhibitors, Finerenone, Type 2 diabetes mellitus, Chronic kidney disease

## Abstract

**Objective:**

To evaluate the efficacy and safety of finerenone and sodium-glucose cotransporter-2 inhibitors (SGLT2i) on reducing new-onset of atrial fibrillation (AF) in patients with type 2 diabetes mellitus (T2DM) and chronic kidney disease (CKD).

**Method:**

We searched the PubMed, Cochrane Library, Web of Science, Medline and Embase covering January 1, 2000 to April 30, 2022. Randomized control trials comparing finerenone or SGLT2i with placebo in patients with T2DM and CKD were selected. Results were reported as risk ratio (RR) with corresponding 95% confidence interval (CI).

**Results:**

A total of 10 studies (35,841 patients) were included. Finerenone (RR 0.79, 95% CI 0.62–0.99) was associated with a decreased risk of AF compared with placebo, while SGLT2i were not. SGLT2i were associated with a decreased risk of hospitalization for heart failure (RR 0.78, 95% CI 0.63–0.98) compared with finerenone. They were comparable in AF(RR 0.84, 95% CI 0.48,1.46), major adverse cardiovascular events(MACE) (RR 0.93, 95% CI 0.81,1.06) and nonfatal stroke(RR 0.78, 95% CI 0.58,1.05). They both showed no significant risk of adverse events compared with placebo.

**Conclusion:**

There was no significant difference in the reduction of new-onset of atrial fibrillation between Finerenone and SGLT2i based on the indirect comparisons of currently available clinical studies. The large-sampled head-to-head trials was needed for the more precise conclusion.

**Supplementary Information:**

The online version contains supplementary material available at 10.1186/s13098-022-00929-3.

## Introduction

As the most common sustained arrhythmia, atrial fibrillation (AF) not only increases the risk of stroke and heart failure, but also leads to the cerebrovascular death [1, 2]. It is generally acknowledged that both chronic kidney disease (CKD) and type 2 diabetes mellitus (T2DM) can induce atrial structural or electrical remodeling through many mechanisms, leading to the development of AF [3–6]. What`s more, morbidity and mortality of AF are very high among patients with diabetes and/or CKD than those without [7, 8]. Treatments such as antihyperglycemic agents, anticoagulant are commonly used based on the patients’ condition, but in the recent year, more medications are available and recognized by professional guidelines, granting patients more options in the essential step of AF prevention.

Finerenone is a nonsteroidal and selective mineralocorticoid receptor antagonist. According to FIDELIO-DKD trial [9], which targeted at T2DM and CKD patients, finerenone can significantly reduce the occurrences of composite cardiovascular outcome, which was defined as a composite of nonfatal myocardial infarction, nonfatal stroke, death from cardiovascular causes, or hospitalization for heart failure (HHF). A secondary analysis of this trial revealed that finerenone could reduce the incidence of new-onset AF in patients with CKD and T2DM [10]. Consequently, in renin–angiotensin–aldosterone system (RAAS) inhibitions, finerenone represents a new frontier in the treatment of diabetic kidney disease [11]. American Diabetes Association (ADA) suggested that in patients with T2DM and CKD who were at increased risk for cardiovascular events or CKD progression or are unable to use a SGLT2i, finerenone was recommended to reduce CKD progression and cardiovascular events [12].

Several large cohort studies and randomized controlled trials (RCTs) have demonstrated favorable cardiovascular outcomes associated with sodium glucose cotransporter-2 inhibitors (SGLT2i) in patients with diabetes or CKD [13–19]. Moreover, two new meta-analyses showed that SGLT2i could provide specific AF-reduction benefits in patients with T2DM and/or CKD [20, 21]. In the light of the existing results of RCTs and meta-analysis, the ADA recommended SGLT2i for individuals with T2DM with or at high risk for atherosclerotic cardiovascular disease, heart failure, and/or CKD [22]. Although the two drugs have completely different mechanisms of action, both have cardiovascular and renal protective effects in patients with CKD and T2DM. Several large RCTs and related meta-analyses have also pointed out that they have the effect of reducing the incidence of new-onset AF in patients with T2DM and CKD. The comparison of the two drug would be useful for practical decision-making by clinicians.

There’s currently a lack of study comparing their effects on reducing AF. The network meta-analysis (NMA) based on indirect and direct comparisons is an efficient method to assist determining the relative cardiovascular efficacy and safety of finerenone and SGLT2i. Therefore, our research aimed to investigate the effectiveness of finerenone and SGLT2i in patients with T2DM and CKD by performing NMA based on RCTs.

## Methods

We prospectively registered this NMA in the International Prospective Register of Systematic Reviews database (PROSPERO) (registration number: CRD42022330769). Our search strategy was conducted in accordance with the Preferred Reporting Items for Systematic Reviews and Meta-Analyses extension statement (PRISMA) [23, 24].

### Data source

We performed a systematic search of PubMed, Cochrane Library, Web of Science, Medline and Embase from January 1, 2000 to April 30, 2022. In order to ensure the comprehensiveness of the retrieval, the combination of subject words and free words were used for literature retrieval.

The pre-specified search keywords were applied as follow: “atrial fibrillation”, “cardiovascular diseases”, “finerenone”, “SGLT2 inhibitors”, “canagliflozin”, “dapagliflozin”, “sotagliflozin”, “empagliflozin”, “ertugliflozin”, “luseoglifozin”, “Diabetes Mellitus, Type 2”, “Renal Insufficiency, Chronic”, “chronic kidney disease”. The detailed search strategies of databases were described in the Additional file 1 Search strategy**.**

### Outcomes

Seven outcomes were assessed in this study, which were divided into primary, secondary and safety outcomes. The primary outcome was the incidence of new-onset AF. The secondary outcomes included major adverse cardiovascular events (MACE), HHF and nonfatal stroke. The safety outcomes included adverse events (AE), serious adverse events (SAE) and serious hyperkalemia (SHK). The definition of MACE was a composite of death from cardiovascular causes, nonfatal myocardial infarction, or nonfatal stroke. If nonfatal myocardial infarction and stroke data were unavailable, the total myocardial infarction and stroke were used instead. The definition of AE was those started or worsened during drugs or placebo intake or up to three days after any temporary or permanent interruption. An adverse event was considered to be serious if it resulted in death, life-threatening accidents, inpatient hospitalization (or prolongation of existing hospitalization), persistent or clinically significant disability or incapacity and congenital abnormality or birth defect.

### Study selection

Studies were selected if they met the following inclusion criteria: (1) published in peer-reviewed journals; (2) included adult patients (≥ 18 years old) with T2DM and(or) CKD; (3) RCTs; (4) compared finerenone or SGLT2i with a placebo; (5) included any of the pre-specified primary and secondary outcomes; (6) written in English. Studies were excluded if they met the following criteria: (1) unavailable data for estimating risk ratio (RR) even after contacting with the authors; (2) unspecified dosage of the intervention drugs; (3) unavailable manuscript.

### Data extraction and quality assessment

The search results were screened separately by two blinded and independent researchers (Z and W) to identify studies according to inclusion and exclusion criteria and with reference to the Cochrane Collaboration Systematic Evaluators manual (version 5.1.0) [25]. When the two authors encountered inconsistencies, a third author (J) was consulted to reach a decision. In addition, we reviewed the list of references included in the meta-analysis studies to minimize missing relevant studies. The duplicate literatures were removed from this study. After that, we used NoteExpress (version 3.6.0) to review the abstract of the remaining literature. The included literature was cross-checked (Z and W), and differences were discussed or judged by a third researcher (J). After thorough discussion, the following data were extracted for further research: demographics, diagnostic criteria, randomization methods, allocation schemes, intervention drugs and dosage, follow-up time and outcome indicators.

Data extraction and risk of bias assessment were performed by two researchers (Z and J), the Cochrane risk of bias assessment tool (RoB 2.0) was applied [26] to conduct the Risk of bias assessment. Should discrepancies appear during assessment or extraction, a third reviewer (W) was responsible for resolution. Targeted data were extracted from each study group. In this study, we applied the Grading of Recommendations Assessment, Development, and Evaluation (GRADE) method, which could be operated in GRADEpro GDT software. The GRADE method allowed us to assess the quality of the evidence for each outcome. They were namely High, Moderate, Low, and Very low. We also referred to these criteria: risk of bias, the inconsistency, the indirectness, the imprecision and the publication bias, in order to prevent any common bias that will alter our result, and leading to the creation of the summary of evidence table [27]. Apart from the criteria above, the intransitivity and incoherence were also taken into consideration. The estimation of the quality of treatment effect was rated based on the quality of direct and indirect comparison strictly adhering to the GRADE Working Group approach [28].

### Statistical analysis

This NMA was performed by using Stata (version 15.0) based on the frequency model. Firstly, the network evidence figure was drawn for each outcome to show the comparison between the two drugs. According to inclusion and exclusion criteria for the literature, only RCTs compared finerenone or SGLT2i with placebo were included, so there was no closed loop between the intervention and only indirect evidence between two drugs. Consequently, there was no need to test inconsistency or rendering incoherence for this NMA. Secondly, we performed forest plot and league table to show the results of NMA. Risk ratio (RR) and 95% confidence interval (CI) were used to present the efficacy of treatments. For dichotomous variables included in this study, treatment associated with reduced RR was considered beneficial. The probability value of the I^2^ variable was calculated to assess heterogeneity, which was considered to be unimportant (0% < I^2^ < 40%), moderate heterogeneity (30% < I^2^ < 60%), substantial heterogeneity (50% < I^2^ < 90%), considerable heterogeneity (75% < I^2^ < 100%) [29]. Finally, we developed a correction funnel plot for each outcome to determine the evidence of small sample effect.

## Results

### Literature search and baseline characteristics of included studies

**Fig. 1:**
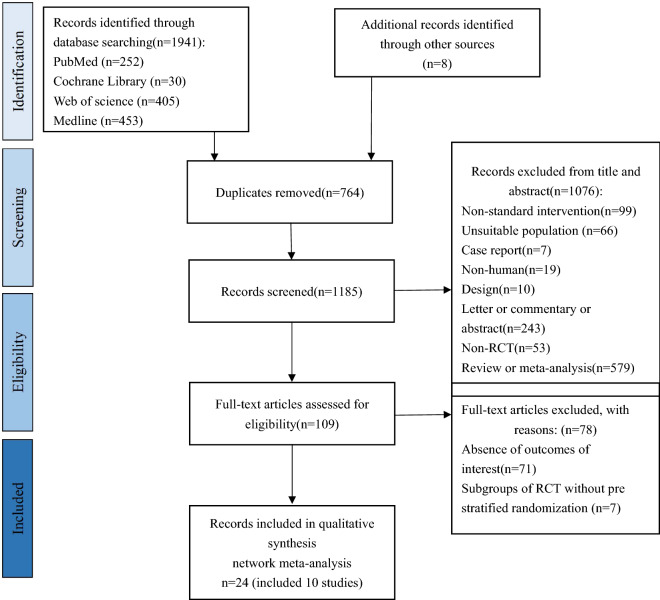
PRISMA flowchart

The detailed study filtering process is shown in Fig. 1. In brief, we retrieved a total of 1941 articles from PubMed (n = 252), Cochrane Library (n = 30), Web of science (n = 405), Medline (n = 453) and Embase (n = 801) in primary search, during the process another 8 articles were identified through references. A total of 764 duplicate articles were removed. After review by title, abstract and assessing full text, 24 articles (included 10 RCTs) were included in this NMA. There were 8 integrated RCTs and 2 RCT subgroups (from EMPA-REG OUTCOME and DAPA-CKD) among all included studies. In the EMPA-REG OUTCOME study, the project's randomization protocol had been stratified according to patients' glomerular filtration rate (≥ 90, 60–89, < 60) and we included data from the subgroup with eGFR < 60 ml/min/1.73 m^2^, so that randomization requirements were still met [13]. Similarly, the DAPA-CKD study stratified patients according to their diabetes status [17]. The subgroup data from these two studies were judged to be valid and appropriate for randomization and were therefore still included in this NMA.

Out of 10 studies, three studies compared finerenone [9, 10, 30–33] with placebo, involved a total of 14,847 patients with T2DM and CKD; 10 studies were compared SGLT2i (Empaglifozin [13, 34–38], Canagliflozin [15, 39–43], Dapagliflozin [17, 44–46], and Sotagliflozin [19, 47]) with placebo, involved a total of 20,994 patients with T2DM and CKD. The characteristics of the included studies are presented in Table 1. The definition of MACE in the included trials was consistent, except for two of them, EMPA-REG (data for nonfatal myocardial infarction and stroke were not available, so we used total myocardial infarction and stroke instead) and FIGARO-DKD (a composite of death from cardiovascular causes, nonfatal myocardial infarction, nonfatal stroke, or HHF).

**Table 1:** Baseline Characteristics of included studies in patients with T2DM and CKD

Trial	Study design	Patients enrolled in trials	Patients enrolled in this study	Intervention (mg/day)	Control	Median follow up (years)	Number of patients			Age		Male		BMI(kg/m^2^)		HbA1C(%)		eGFR (ml/min/1.73 m^2^)		Duration of diabetes(years)	
							Total	I	C	I	C	I	C	I	C	I	C	I	C	I	C
Finerenone vs placebo																					
FIDELIO-DKD [9, 10, 30, 31]	RCT	T2D and CKD	T2D and CKD	Finerenone 10/20	Placebo	2.6	6674	2833	3841	65.4 ± 8.9	65.7 ± 9.2	1953	2030	N/A		7.7 ± 1.3	7.7 ± 1.4	44.4 ± 12.5	44.3 ± 12.6	16.6 ± 8.8	16.6 ± 8.8
FIGARO-DKD [30, 32]	RCT	T2D and CKD	T2D and CKD	Finerenone 10/20	Placebo	3.4	7352	3686	3666	64.1 ± 9.8		5107		31.4 ± 6.0		7.7 ± 1.4		67.8 ± 21.7		14.5 ± 8.5	
ARTS-DN [33]	RCT	DN	DN	Finerenone 1.25–20	Placebo	0.2	821	727	94	64.33 ± 9.20	63.26 ± 8.68	570	69	31.75 ± 5.57	32.49 ± 5.27	7.6 ± 1.3	7.6 ± 1.3	66.9 ± 21.9	72.2 ± 20.4	N/A	
SGLT2i vs placebo																					
EMPA-REG OUTCOME [13, 34–37]	RCT	T2D	T2D and CKD	Empagliflozin 10/25	Placebo	3.1	1819	1212	607	67.1 ± 7.6	67.1 ± 8.2	816	418	31.0 ± 5.5	30.9 ± 5.4	8.07 ± 0.86	8.03 ± 0.85	48.4 ± 8.2	48.6 ± 7.8	N/A	
EMPA-REG RENAL [38]	RCT	T2D and CKD	T2D and CKD	Empagliflozin 10/25	Placebo	1.0	738	419	319	63.8 ± 8.8	64.1 ± 8.7	249	181	30.9 ± 5.5	30.7 ± 5.5	8.00 ± 0.83	8.10 ± 0.82	55.7 ± 18.8	49.9 ± 18.7	N/A	
CREDENCE [15, 39–42]	RCT	T2D and CKD	T2D and CKD	Canagliflozin 100	Placebo	2.6	4401	2202	2199	62.9 ± 9.2	63.2 ± 9.2	1440	1467	31.4 ± 6.2	31.3 ± 6.2	8.3 ± 1.3	8.3 ± 1.3	56.3 ± 18.2	56.0 ± 18.3	15.5 ± 8.7	16.0 ± 8.6
NCT01064414 [43]	RCT	T2D and CKD	T2D and CKD	Canagliflozin 100/300	Placebo	1.0	269	179	90	68.7 ± 8.2	68.2 ± 8.4	106	57	32.9 ± 6.0	33.1 ± 6.5	7.9 ± 0.9	8.0 ± 0.9	39.1 ± 6.9	40.1 ± 6.8	16.3 ± 7.6	16.4 ± 10.1
DAPA-CKD [17, 44–46]	RCT	CKD	T2D and CKD	Dapagliflozin 10	Placebo	2.4	2906	1455	1451	64.1 ± 9.8	64.7 ± 9.5	961	980	30.2 ± 6.2	30.4 ± 6.3	7.8 ± 1.7	7.8 ± 1.6	44.0 ± 12.6	43.6 ± 12.6	13.7	13.8
SCROED [19]	RCT	T2D and CKD	T2D and CKD	Sotagliflozin 400	Placebo	1.3	10,584	5292	5292	69	69	2945	2885	31.9	31.7	8.3	8.3	44.4	44.7	N/A	
NCT03242018[47]	RCT	T2D and CKD	T2D and CKD	Sotagliflozin 200/400	Placebo	1.0	277	184	93	67.1 ± 9.8	68.0 ± 8.3	93	42	31.5 ± 5.8	31.7 ± 5.7	8.3 ± 0.9	8.4 ± 1.1	23.9 ± 4.6	24.1 ± 4.4	19.1 ± 9.2	20.7 ± 8.9

I: intervention; C: control; DN: diabetic nephropathy; n/a: not available

### Risk of bias and GRADE assessment

**Fig. 2:**
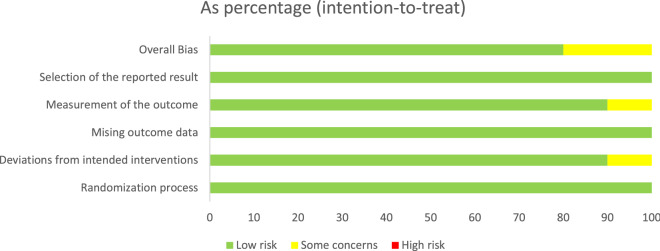
Risk of bias

Among the 10 included RCTs, two were evaluated as “some concerns” for deviations from intended interventions and the outcome assessors were not blinded to intervention status, while the other 8 RCTs were found to present low risk of bias. The proportion of each item was shown in Fig. 2, the quality evaluation of each included studies is shown in Additional file 2 ROB-2 evaluation.

In each of the seven terms we focused on, there were two direct contrasts in the original articles. In terms of reducing the incidence of new-onset AF, the comparison between finerenone and placebo had statistically significant results, which was rated as moderate. In terms of reducing the incidence of MACE and HHF, both comparisons had statistically significant results, which estimated results were moderate in HHF and high in MACE. In terms of reducing the incidence of nonfatal stroke, the comparisons between SGLT2i and placebo had statistically significant result, which estimated result was moderate. In terms of reducing the incidence of SAE, both comparisons had statistically significant results, which estimated result was moderate (SGLT2i vs placebo) and high (finerenone vs placebo). The detail is shown in Table 2.

**Table 2:** GRADE assessment

Certainty assessment							№ of patients		Certainty	Importance
Intervention of studies	Study design	Risk of bias	Inconsistency	Indirectness	Imprecision	Publication bias	Intervention group	Control group		
Incidence of new-onset AF (No of studies: 7)										Critical
SGLT2i vs Placebo	Randomised trials	Serious	Not serious	Not serious	Serious	None	25/2984 (0.8%)	26/2701 (1.0%)	Low	
Finerenone vs Placebo	Randomised trials	Not serious	Not serious	Not serious	Serious	None	123/7246 (1.7%)	155/6601 (2.3%)	Moderate	
Major adverse cardiovascular events (No of studies: 10)										Important
SGLT2i vs Placebo	Randomised trials	Not serious	Not serious	Not serious	Not serious	None	856/10345 (8.3%)	940/9642 (9.7%)	High	
Finerenone vs Placebo	Randomised trials	Not serious	Not serious	Not serious	Not serious	None	686/6519 (10.5%)	777/6507 (11.9%)	High	
Hospitalization for heart failure (No of studies: 10)										Important
SGLT2i vs Placebo	Randomised trials	Not serious	Not serious	Not serious	Serious	None	281/10345 (2.7%)	410/9642 (4.3%)	Moderate	
Finerenone vs Placebo	Randomised trials	Not serious	Not serious	Not serious	Serious	None	256/6519 (3.9%)	325/6507 (5.0%)	Moderate	
Nonfatal stroke (No of studies: 8)										Important
SGLT2i vs Placebo	Randomised trials	Not serious	Not serious	Not serious	Serious	None	146/8890 (1.6%)	158/8191 (1.9%)	Moderate	
Finerenone vs Placebo	Randomised trials	Not serious	not serious	not serious	Serious	None	198/6519 (3.0%)	198/6507 (3.0%)	Moderate	
Adverse events (No of studies:6)										Important
SGLT2i vs Placebo	Randomised trials	Serious	Not serious	Not serious	Not serious	Suspected	5807/7857 (73.9%)	5758/7674 (75.0%)	Low	
Finerenone vs Placebo	Randomised trials	Not serious	Not serious	Not serious	Not serious	None	6035/7246 (83.3%)	5654/6601 (85.7%)	High	
Serious adverse events (No of studies:10)										Important
SGLT2i vs Placebo	Randomised trials	Serious	Not serious	Not serious	Not serious	None	631/13197 (4.8%)	597/11689 (5.1%)	Moderate	
Finerenone vs Placebo	Randomised trials	Not serious	Not serious	Not serious	Not serious	None	2095/7246 (28.9%)	2189/6601 (33.2%)	High	
Serious hyperkalemia (No of studies:4)										Important
SGLT2i vs Placebo	Randomised trials	Not serious	Not serious	Not serious	Serious	None	10/2202 (0.5%)	10/2199 (0.5%)	Moderate	
Finerenone vs Placebo	Randomised trials	Not serious	Not serious	Not serious	Serious	None	74/7246 (1.0%)	16/6601 (0.2%)	Moderate	

According to recommendation of GRADE working group, we presented a four-step approach to rate the quality of evidence in each of the direct, indirect, and network meta-analysis estimates based on methods developed by the GRADE working group [28]. The baseline eGFR of patients in the “NCT03242018” was different from other studies. For direct comparisons, “NCT03242018” included only 1.3% of patients in SGLT2i (277/20,994). Therefore, risk of bias was not taken in to consideration. As for intransitivity, there was only indirect evidence in the intercomparison of the two drugs. The GRADE working group recommends in such situation issues regarding intransitivity may warrant particular attention, and the threshold for rating down for intransitivity may be lower [28]. Therefore, we downgraded the quality of evidence for the comparison between finerenone and SGLT2i. The detail of GRADE assessment is shown in Table 2. Estimates of effects and quality ratings for comparison of drugs is shown in Table 3.

**Table 3:** Estimates of effects and quality ratings for comparison of drugs

Comparison	Direct evidence		Indirect evidence		Network meta-analysis	
	RR [95% CI]	Quality of evidence	RR [95% CI]	Quality of evidence	RR [95% CI]	Quality of evidence
Incidence of new-onset AF						
SGLT2i vs Placebo	0.85 (0.50,1.47)	Low*^■^	Not estimable^▲^	–	0.84 (0.48,1.46)	Low*^■^
Finerenone vs Placebo	0.79 (0.62,0.99)	Moderate^■^	Not estimable^▲^	–	0.79 (0.62,0.99)	Moderate^■^
SGLT2i vs Finerenone	–	–	1.06 (0.58,1.94)	Very Low ^●^	1.06 (0.58,1.94)	Very Low ^●^
Major adverse cardiovascular events						
SGLT2i vs Placebo	0.81 (0.74,0.89)	High	Not estimable^▲^	–	0.81 (0.75,0.89)	High
Finerenone vs Placebo	0.88 (0.80,0.97)	High	Not estimable^▲^	–	0.88 (0.80,0.97)	High
SGLT2i vs Finerenone	–	–	0.93 (0.81,1.06)	Moderate^●^	0.93 (0.81,1.06)	Moderate^●^
Hospitalization for heart failure						
SGLT2i vs Placebo	0.62 (0.53,0.72)	Moderate^■^	Not estimable^▲^	–	0.62 (0.53,0.72)	Moderate^■^
Finerenone vs Placebo	0.79 (0.67,0.92)	Moderate^■^	Not estimable^▲^	–	0.79 (0.67,0.92)	Moderate^■^
SGLT2i vs Finerenone	–	–	0.78 (0.63,0.98)	Low^●^	0.78 (0.63,0.98)	Low^●^
Nonfatal stroke						
SGLT2i vs Placebo	0.77 (0.62,0.97)	Moderate^■^	Not estimable^▲^	–	0.78 (0.62,0.97)	Moderate^■^
Finerenone vs Placebo	1.00 (0.82,1.21)	Moderate^■^	Not estimable^▲^	–	1.00 (0.82,1.21)	Moderate^■^
SGLT2i vs Finerenone	–	–	0.78 (0.58,1.05)	Low^●^	0.78 (0.58,1.05)	Low^●^
Adverse events						
SGLT2i vs Placebo	0.98 (0.96,1.00)	Low*^▷^	Not estimable^▲^	–	0.98 (0.96,1.00)	Low*^▷^
Finerenone vs Placebo	1.00 (0.99,1.01)	Moderate^▷^	Not estimable^▲^	–	1.00 (0.98,1.01)	Moderate^▷^
SGLT2i vs Finerenone	–	–	0.99 (0.96,1.01)	Very Low^●^	0.98 (0.96,1.00)	Very Low^●^
Serious adverse events						
SGLT2i vs Placebo	0.89 (0.81,0.99)	Moderate*	Not estimable^▲^	–	0.90 (0.86,0.93)	Moderate*
Finerenone vs Placebo	0.94 (0.90,0.99)	High	Not estimable^▲^	–	0.94 (0.90,0.99)	High
SGLT2i vs Finerenone	–	–	0.95 (0.89,1.02)	Low^●^	0.95 (0.89,1.02)	Low^●^
Serious hyperkalemia						
SGLT2i vs Placebo	1.00 (0.42,2.39)	Moderate^■^	Not estimable^▲^	–	1.00 (0.42,2.39)	Moderate^■^
Finerenone vs Placebo	4.16 (2.45,7.08)	Moderate^■^	Not estimable^▲^	–	4.08 (2.39,6.96)	Moderate^■^
SGLT2i vs Finerenone	–	–	0.25 (0.09,0.68)	Low^●^	0.25 (0.09,0.68)	Low^●^

*Risk of bias, ^■^Imprecision, ^▷^Publication bias, ^▲^Cannot be estimated because the drug was not connected in a loop in the evidence network, ^●^Intransitivity

### NMA results of the primary outcome

**Fig. 3:**
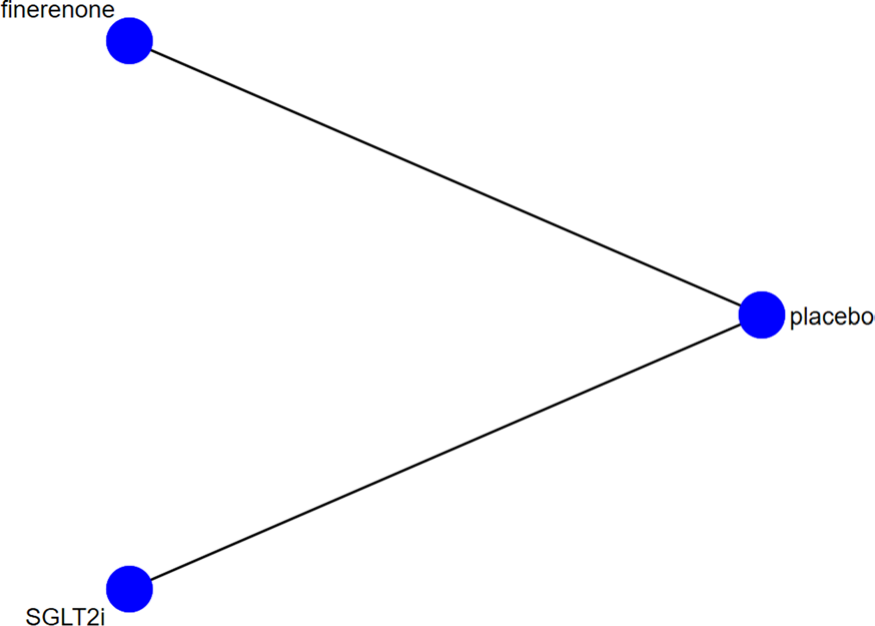
Network evidence figure

**Fig. 4:**
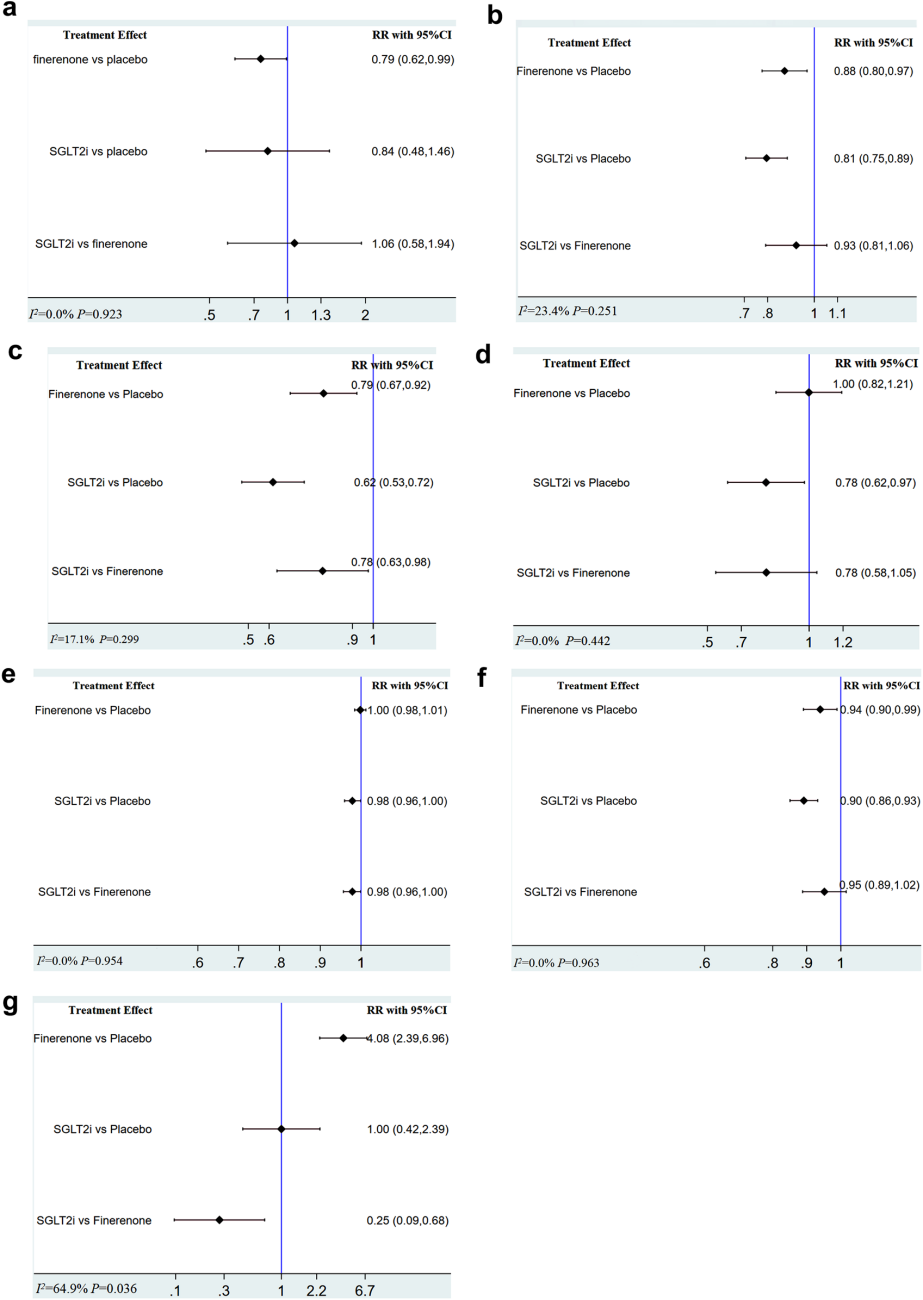
**a** NMA reporting RR for primary outcome in patients with T2DM and CKD. **b** NMA reporting RR for major adverse cardiovascular events in patients with T2DM and CKD. **c** NMA reporting RR for hospitalization for heart failure in patients with T2DM and CKD. **d** NMA reporting RR for nonfatal stroke in patients with T2DM and CKD. **e** NMA reporting RR for adverse events in patients with T2DM and CKD. **f** NMA reporting RR for serious adverse events in patients with T2DM and CKD. **g** NMA reporting RR for serious hyperkalemia in patients with T2DM and CKD

Figure 3 shows the network graph. For the primary outcome, SGLT2i didn`t show a significant effect in reducing the incidence of AF compared with placebo (RR 0.84, 95% CI 0.48–1.46), but finerenone (RR 0.79, 95% CI 0.62–0.99) was associated with a decreased risk of AF. There was also no significant difference between SGLT2i and finerenone (RR 1.06, 95% CI 0.58–1.94). There was no heterogeneity (I^2^ = 0.0%, p = 0.923). The detail is shown in Fig. 4a.

### NMA results of the secondary outcome

For the secondary outcome MACE, both finerenone (RR 0.88, 95% CI 0.80–0.97) and SGLT2i (RR 0.81, 95% CI 0.75–0.89) were shown to be significantly more effective compared with placebo. And these two kinds of drugs were comparable (RR 0.93, 95% CI 0.81–1.06). There was unimportant heterogeneity (I^2^ = 23.4%, p = 0.251). The detail is shown in Fig. 4b. As for HHF, both finerenone (RR 0.79, 95% CI 0.67–0.92) and SGLT2i (RR 0.62, 95% CI 0.53–0.72) were associated with a lower risk compared with placebo. SGLT2i (RR 0.78, 95% CI 0.63–0.98) significantly decreased the risk of HHF compared with finerenone. There was unimportant heterogeneity (I^2^ = 17.1%, p = 0.299). The detail is shown in Fig. 4c. When it comes to nonfatal stroke, SGLT2i (RR 0.78, 95% CI 0.62–0.97) were shown to be significantly more effective than placebo, while finerenone was not (RR 1.00, 95% CI 0.82–1.21). SGLT2i and finerenone were comparable (RR 0.78, 95% CI 0.58–1.05) in reducing the incidence of nonfatal stroke. There was unimportant heterogeneity (I^2^ = 0.0%, p = 0.442). The detail is shown in Fig. 4d.

### NMA results of the safety outcome

For the safety outcome AE, SGLT2i had a tendency to decrease the incidence of AE compared with placebo (RR 0.98, 95% CI 0.96–1.00). And finerenone showed no significant risk of AE compared with SGLT2i (RR 0.98, 95% CI 0.96–1.00) or placebo (RR 1.00, 95% CI 0.98–1.01). There was no heterogeneity (I^2^ = 0.0%, p = 0.954). The detail is shown in Fig. 4e. When it comes to SAE, both SGLT2i (RR 0.90, 95% CI 0.86–0.93) and finerenone (RR 0.94, 95% CI 0.90–0.99) were associated with a lower risk of SAE compared with placebo. And these two kinds of drugs were comparable (RR 0.95, 95% CI 0.89–1.02). There was no heterogeneity (I^2^ = 0.0%, p = 0.963). The detail is shown in Fig. 4f. As for SHK, SGLT2i (RR 0.25, 95% CI 0.09–0.68) were shown to be more effective than finerenone. And SGLT2i (RR 1.00, 95% CI 0.42–2.39) showed no significant risk of AE compared with placebo, while finerenone (RR 4.08, 95% CI 2.39–6.96) was not. There was substantial heterogeneity (I^2^ = 64.9%, p = 0.036). The detail is shown in Fig. 4g.

### Publication bias and heterogeneity

As is shown in Additional file 3 Publication bias, for the six outcomes (incidence of new-onset AF, MACE, HHF, nonfatal stroke, SAE and SHK), all the studies were distributed symmetrically on both sides of the midline. However, for the efficacy outcome AE, some studies were not completely symmetrically distributed on both sides of the midline, and the corrected regression line was almost parallel to the X axis. Therefore, it is suggested that publication bias and small sample effect may exist for AE, while the possibility of the other six outcomes indicators are very small. We only observed substantial heterogeneity in the safety outcome of AE, the reason may be related to the treatment and follow-up time of the drugs and the number of patients included in different studies.

## Discussion

The comparison between SGLT2i and finerenone on the risk of AF in patients with CKD and T2DM remained unclear in the absence of direct RCTs. This NMA evaluated the relative efficacy and safety of two drugs on reducing new-onset of AF in patients with T2DM and CKD. Our NMA was based on 10 studies, which included 35,841 patients randomly assigned to finerenone or SGLT2i or placebo.

Our results revealed that finerenone could decrease the incidence of AF in patients with T2DM and CKD, but SGLT2i could not. Such results varied with another recent NMA [21]. The cause of such phenomenon may be that they only included three trials correlating to SGLT2i (DECLARE-TIMI 58, CANVAS Program and CREDENCE), Furthermore, that paper only included data from a subgroup of patients with combined CKD in DECLARE-TIMI 58 and the CANVAS Program, which did not follow a strict randomization process and therefore did not have a high level of evidence.

SGLT2i and finerenone were equivalent on reducing the risk of AF. As for HHF and nonfatal stroke, SGLT2i were better compared to finerenone. Our study found the advantage of finerenone in reducing the risk of MACE and HHF, SGLT2i have benefits in reducing nonfatal stroke. Such results varied with another recent NMA [48]. The cause of such phenomenon may be that they only included one trial correlating to finerenone (FIDELIO-DKD) and they failed to include the results of EMPA-REG OUTCOME trial on nonfatal stroke. These findings suggested that for those who are susceptible to AF, finerenone may have a potential risk reduction advantage over SGLT2i (RR 0.84). But there was no significant difference between the two drugs (CI 0.48,1.46). It was also noted that SGLT2i outperformed finerenone when it comes to reducing the risk of HHF and nonfatal stroke.

As for the safety outcomes, neither SGLT2i nor finerenone showed a significant advantage or disadvantage in reducing AE. Our results showed that the safety of the two drugs in AE and SAE were approximately equivalent. Although finerenone has been shown smaller effects on serum potassium levels than spironolactone [49, 50], it was still associated with increased risk of SHK due to the antagonism of aldosterone receptors.

Several mechanisms have been proposed for the positive impact of finerenone. Finerenone has been shown to attenuate adverse atrial remodeling related to CKD or T2DM, by inhibiting aldosterone activity, such as the prevention of fibrotic remodeling of the atrial myocardium via interfering with the small GTPase Rac1, limiting aldosterone/mineralocorticoid receptor-induced expression of the key profibrotic mediator connective tissue growth factor, and the collagen crosslinking enzyme lysyl oxidase, as well as microRNA-21, which enhances myocardial remodeling and fibrosis [5, 6, 51–54]. Furthermore, finerenone significantly reduced mineralocorticoid receptor overactivation-mediated protein expression of transforming growth factor-beta and collagen 3 alpha 1, as well as fibrosis in transgenic mice with cardiac-specific overexpression of Rac1 [52], which may explain its benefits.

As for the nonfatal stroke and HHF, it was clear that SGLT2i had more significant impact than finerenone. Unlike finerenone, SGLT2i had the natriuretic and diuretic effect, it could improve renal ultrafiltration and hypoxia and thus reduce blood glucose, oxidative stress, body weight, uric acid, and blood pressure [55–63]. Interestingly our results showed that SGLT2i could not reduce the incidence of AF in patients with T2DM and CKD, but it could significantly reduce the risk of HHF. At present, many studies have found that the AF-reduction effects of SGLT2i may be partly independent of heart failure improvement, and the pharmacological effects on ameliorating cardiac fibrosis caused by AF appears to be different from those in heart failure. Although the specific mechanism is unknown, this could be one of the reasons for this result. Another reason why SGLT2i were effective in patients with T2DM while ineffective in patients with T2DM and CKD, could be the mechanism of AF caused by CKD is different than diabetes, as AF in diabetes may be caused by increased reactive oxygen species or advanced glycation end products [5, 6].

## Strengths and limitations

A major strength of this NMA is that this is the first study which investigates the effect of finerenone and SGLT2i on reducing new-onset of AF in patients with T2DM and CKD. Second, the statistical efficiency was relatively reliable, because the number of studies and sample size we included were large enough, and most clinical studies were high-quality RCTs.

However, some limitations existed. Firstly, only indirect comparisons between two drugs were included, our results require validation by head-to-head trials. Secondly, partial RCTs included in this NMA were rated as low quality, both ranking results and clinical application should be carefully examined based on real world circumstances, expert opinion and guidelines. Lastly, there were more patients involved in SGLT2i than finerenone. Although considerable heterogeneity was not observed, there was substantial heterogeneity in one of the safety outcomes and these imbalances may hinder the statistical capabilities of NMA.

## Conclusions

In patients with T2DM and CKD, SGLT2i may not have benefits on reducing new-onset of AF, although SGLT2i are more effective than finerenone in reducing HHF and nonfatal stroke. Both SGLT2i and finerenone have lower risk of MACE and SAE, and they were approximately comparable in safety outcomes. However, further validation by head-to-head trials comparing finerenone with SGLT2i would be beneficial. Moreover, there is a need for further studies to assess whether SGLT2i have different effect on reducing new-onset of AF in patients with T2DM and CKD and those without CKD.

## Supplementary Information

**Additional file 1**. Search strategies.

**Additional file 2.** ROB-2 evaluation.

**Additional file 3. **Publication bias.

## Data Availability

All data generated or analyzed during this study are included in this published article.

## Abbreviations

AF: Atrial fibrillation
CKD: Chronic kidney disease
T2DM: Type 2 diabetes mellitus
RCTs: Randomized controlled trials
SGLT2i: Sodium glucose cotransporter-2 inhibitors
NMA: Network meta-analysis
MACE: Major adverse cardiovascular events
HHF: Hospitalization for heart failure
AE: Adverse events
SAE: Serious adverse events
SHK: Serious hyperkalemia
GRADE: Grading of Recommendations Assessment, Development, and Evaluation
RR: Risk ratio
CI: Confidence interval
