# Supplementary material for: Network meta-analysis on the efficacy and safety of finerenone versus SGLT2 inhibitors on reducing new-onset of atrial fibrillation in patients with type 2 diabetes mellitus and chronic kidney disease
Source: Diabetol Metab Syndr. 2022 Oct 27;14:156. doi: 10.1186/s13098-022-00929-3 (PMC9609222; doi:10.1186/s13098-022-00929-3)
Supplement: Supplementary file 1 — Additional file 1. Search strategies. [file 13098_2022_929_MOESM1_ESM.docx]

**Additional file 1 Search strategies**

Database: PubMed <January 1,2000 to April 30, 2022>

#1 (Diabetes Mellitus, Type 2[MeSH Terms]) OR (Diabetes Mellitus, Type 2[Title/Abstract]) OR (T2DM[Title/Abstract]) OR (type 2 diabetes mellitus[Title/Abstract]) OR (T2D[Title/Abstract])

#2 (atrial fibrillation[MeSH Terms]) OR (Cardiovascular Diseases[MeSH Terms]) OR (atrial fibrillation[Title/Abstract]) OR (atrial flutter[Title/Abstract])

#3 (Renal Insufficiency, Chronic[MeSH Terms]) OR (chronic kidney disease[Title/Abstract]) OR (CKD[Title/Abstract])

#4 (Finerenone[Title/Abstract])

#5 (SGLT-2 inhibitor[Title/Abstract]) OR (SGLT-2[Title/Abstract]) OR (SGLT2[Title/Abstract]) OR (Canagliflozin[Title/Abstract]) OR (Dapagliflozin[Title/Abstract]) OR (sotagliflozin[Title/Abstract]) OR (empagliflozin[Title/Abstract]) OR (Ertugliflozin[Title/Abstract]) OR (luseoglifozin[Title/Abstract]) OR (Canagliflozin[MeSH Terms])

#6 #1 AND #2 AND #3

#7 #4 OR #5

#8 #6 AND #7

#9 #8 AND (2000/1/1:2022/4/30[pdat]) AND (english[Filter])

Database: Web of Science <January 1,2000 to April 30, 2022>

#1 TS=(Diabetes Mellitus, Type 2) OR TS=(T2D) OR TS=(DM) OR TS=(T2DM) OR TS=(type 2 diabetes mellitus)

#2 ALL=(atrial fibrillation) OR ALL=(atrial flutter) OR TS=(Cardiovascular Disease)

#3 ALL=(Finerenone)

#4 TS=(Sodium-Glucose Transporter 2 Inhibitors) OR TS=(SGLT-2 inhibitor) OR TS=(SGLT-2) OR TS=(SGLT2) OR TS=(Canagliflozin) OR TS=(Dapagliflozin) OR TS=(sotagliflozin) OR TS=(empagliflozin) OR TS=(Ertugliflozin) OR TS=(luseoglifozin)

#5 TS=(Renal Insufficiency, Chronic) OR TS=(chronic kidney disease) OR TS=(CKD)

#6 #4 OR #3

#7 #5 AND #2 AND #6 AND #1

#8 #7 AND (2000/1/1:2022/4/30[pdat])

#9 #8 and English (Languages)

Database: Medline <January 1,2000 to April 30, 2022>

#1 TS=(Diabetes Mellitus, Type 2) OR TS=(T2D) OR TS=(DM) OR TS=(T2DM) OR TS=(type 2 diabetes mellitus)

#2 TS=(atrial fibrillation) OR TS=(atrial flutter) OR TS=(Cardiovascular Disease)

#3 TS=(Finerenone)

#4 TS=(Sodium-Glucose Transporter 2 Inhibitors) OR TS=(SGLT-2 inhibitor) OR TS=(SGLT-2) OR TS=(SGLT2) OR TS=(Canagliflozin) OR TS=(Dapagliflozin) OR TS=(sotagliflozin) OR TS=(empagliflozin) OR TS=(Ertugliflozin) OR TS=(luseoglifozin)

#5 TS=(Renal Insufficiency, Chronic) OR TS=(chronic kidney disease) OR TS=(CKD)

#6 #4 OR #3

#7 #5 AND #2 AND #6 AND #1

#8 #7 AND (2000/1/1:2022/4/30[pdat])

#9 #8 and English (Languages)

Database: Cochrane Library <January 1,2000 to April 30, 2022>

**#1**  finerenone[all text]

**#2** MeSH descriptor:[Diabetes Mellitus, Type 2] explode all trees

#3 MeSH descriptor:[Sodium-Glucose Transporter 2 Inhibitors] explode all trees

#4 dapagliflozin [all text]

#5 sotagliflozin [all text]

#6 empagliflozin [all text]

#7 MeSH descriptor:[Canagliflozin] explode all trees

#8 ertugliflozin [all text]

#9 luseoglifozin [all text]

#10 MeSH descriptor:[Atrial Fibrillation] explode all trees

#11 MeSH descriptor:[ Atrial Flutter] explode all trees

#12 MeSH descriptor:[ Cardiovascular Disease ] explode all trees

#13 MeSH descriptor:[ Renal Insufficiency, Chronic ] explode all trees

#14 #1 or #3 or #4 or #5 or #6 or #7 or #8 or #9

#15 #10 or #11 or #12

#16 #2 or #13 or #15

#17 #14 or #16 and (2000/1/1:2022/4/30[pdat])

Database: Embase<January 1,2000 to April 30, 2022>

#1 ‘chronic kidney failure’/exp AND‘non insulin dependent diabetes mellitus’/exp

#2 'sodium glucose cotransporter 2 inhibitor'/exp OR 'dapagliflzon' OR 'satagliflozin' OR 'empagliflozin'/exp OR 'canagliflozin'/exp OR ertugliflozin:ab,ti OR luseogliflozin:ab,ti

#3 'finerenone'/exp

#4 'heart atrium flutter'/exp OR 'atrial fibrillation'/exp OR 'cardiovascular disease'/exp

#5 #2 OR #3

#6 #1 AND #4 AND #5

#7 #1 AND #4 AND #5 AND [english]/lim AND [01-01-2000]/sd NOT [01-05-2022]/sd
